# A Prospective Screening of HLA-B*57.01 Allelic Variant for Preventing the Hypersensivity Reaction to Abacavir: Experience from the Laboratory of Molecular Biology of the Infectious Diseases Division at the University Hospital of Salerno

**Published:** 2014-12-19

**Authors:** C Senatore, B Charlier, A Truono, R Punzi, F D’Aniello, N Boffa, V Izzo, V Conti, G Russomanno, V Manzo, A Filippelli, M. Mazzeo

**Keywords:** Abacavir, HIV, HLA-B*57.01, hypersensitivity reaction, Real-Time PCR

## Abstract

Abacavir is a nucleoside reverse transcriptase inhibitor largely used as part of the antiretroviral therapy in Human Immunodeficiency Virus (HIV)-infected patients. Some individuals (2–9%) who start an abacavir treatment show an immunologic reaction indicated as hypersensitivity reaction syndrome (HSR) that is often responsible for therapy discontinuation and could represent a life-threatening event. Some studies demonstrated a correlation between this adverse reaction and the class I of the major histocompatibility complex (MHC) allele, HLA-B*57.01, in several populations, including Caucasians. Nowadays, International HIV treatment guidelines recommend the HLA-B*57.01 genotyping before abacavir administration to reduce the incidence of HSR. Both male and female HIV-infected patients were enrolled at the Infectious Diseases Division at the University Hospital of Salerno, and admitted to a prospective HLAB*57.01 screening. Genetic analysis was carried out through two sequential Real-Time PCR reactions in which Sybr-Green was used. Out of 248 patients, 215 were Italians from Southern Italy and 33 were coming from several non-EU members countries. All were genotyped: 6 Italians (2.8%) and 1 of the non-EU group (3%) were identified as HLAB*57.01 carriers. In this paper we present our experience in the field of abacavir pharmacogenetic and confirm the importance of Real Time PCR as a valid and cost-effective HLA-B*57.01 typing methodology.

## I. INTRODUCTION

According to the international HIV treatment guidelines, antiretroviral therapy (ART) is recommended for Human Immunodeficiency Virus (HIV)-infected individuals to reduce the risk of disease progression and transmission [1], and abacavir (ABC) is currently used as part of the combination therapy. Unfortunately, many drugs used for the ART are responsible for severe hypersensitivity reactions (HSRs) and ABC is not an exception [2]. Abacavir is a nucleoside reverse transcriptase inhibitor (NRTI) highly active against HIV. ABC is generally well tolerated but several adverse effects, most of all the HSR, affect its clinical use. ABC hypersensitivity reaction (ABCHSR) typically involves skin reactions but is also characterized by symptoms, such as fever, myalgia and gastrointestinal disorders. Moreover, the involvement of internal organs with occurrence of hepatitis, myocarditis, nephritis etc. has been also described. Regardless of the symptoms, ABC-HSR affects 2–9% of patients initiating the treatment and depends, at least in part, on the genetic background of patients [3]. Some studies have described a relationship between ABC-HSR and the class I of the major histocompatibility complex (MHC) allele, HLA-B*57.01 in several populations [4–6] and, more recently, a large double blind, prospective randomized trial (PREDICT-1 study) has confirmed such association [7]. Currently, the Food and Drug Administration (FDA), the US Department of Health and Human Services and the European Medicines Agency (EMA) suggest to adopt an antiretroviral agent alternative to ABC for patients bearing the HLA-B*57.01 allele (HLA-B*57.01-positive). Several molecular methodologies for the screening of HLA-B*57.01 have been validated so far. Here we show the results from an HLA-B*57.01 prospective screening, aimed at preventing the ABC-HSR, in which Real-Time PCR/melting curve with Sybr-Green was used.

## II. METHODOLOGY

Both female and male HIV-infected patients were admitted to a prospective HLA-B*57.01 screening at the Laboratory of Molecular Biology of the Infectious Diseases Division at the University Hospital of Salerno to help reducing the incidence of ABC-HSR. None of the patients had previously been treated with abacavir.

Whole blood samples were collected in K2-EDTA-treated tubes (BD, Milan-Italy) and genomic DNA was isolated through MagMax 96 DNA multi Sample Kit (Life Tech. Corp.) according to the manufacturer’s instructions. DNA concentration was measured using the Nano Photometer (IMPLEN, Munich-Germany) and samples were stored at −20°C until analysis.

HLA-B*57.01 genotyping was performed by Real Time PCR with Sybr-Green, followed by a melting curve determination; the reaction was arranged in two sequential steps. First, we detected the presence of major histocompatibility class (MHC)-I allotype, B*57. The PCR reaction contained 50 ng of total DNA, 2x Sybr-Green Master Mix (Life Tech. Corp.), 100nM of each primer (1F and 4R) in a 25 µl final volume. After initial denaturation of 10 min at 95°C, a total of 40 PCR cycles were performed using the following conditions (95°C for 15 s and annealing/extension at 60°C for 1 min). The second PCR reaction was carried out to identify B*57.01 carriers in B*57 positive samples previously validated by melting curve analysis (~Tm 87.1 °C). In this case, the PCR reaction contained 25ngof total DNA, 2x Sybr-Green Master Mix (Life Tech. Corp.), 200nM of each primer (indicated as 1F, 2R and 3R) in a 25 µl final reaction volume; the conditions used were the same as described for the first PCR. Data from the second PCR reaction were verified by melting curve analysis (~Tm 85.1°C). PCR reactions were performed using an Applied Biosystems (ABI) 7500 Real-time PCR System (Life Tech. Corp.). Sequence-specific primers were synthesized according to the sequence reported by Martin et al. [8]:

**1F**-GTCTCACATCATCCAGGT;
**2/3R**-CCGCCTACGACGGCAAGGAT;
**4R**- CCCAGCGCAAGTGGGAGGCG.

## III. RESULTS

**Figure 1:**
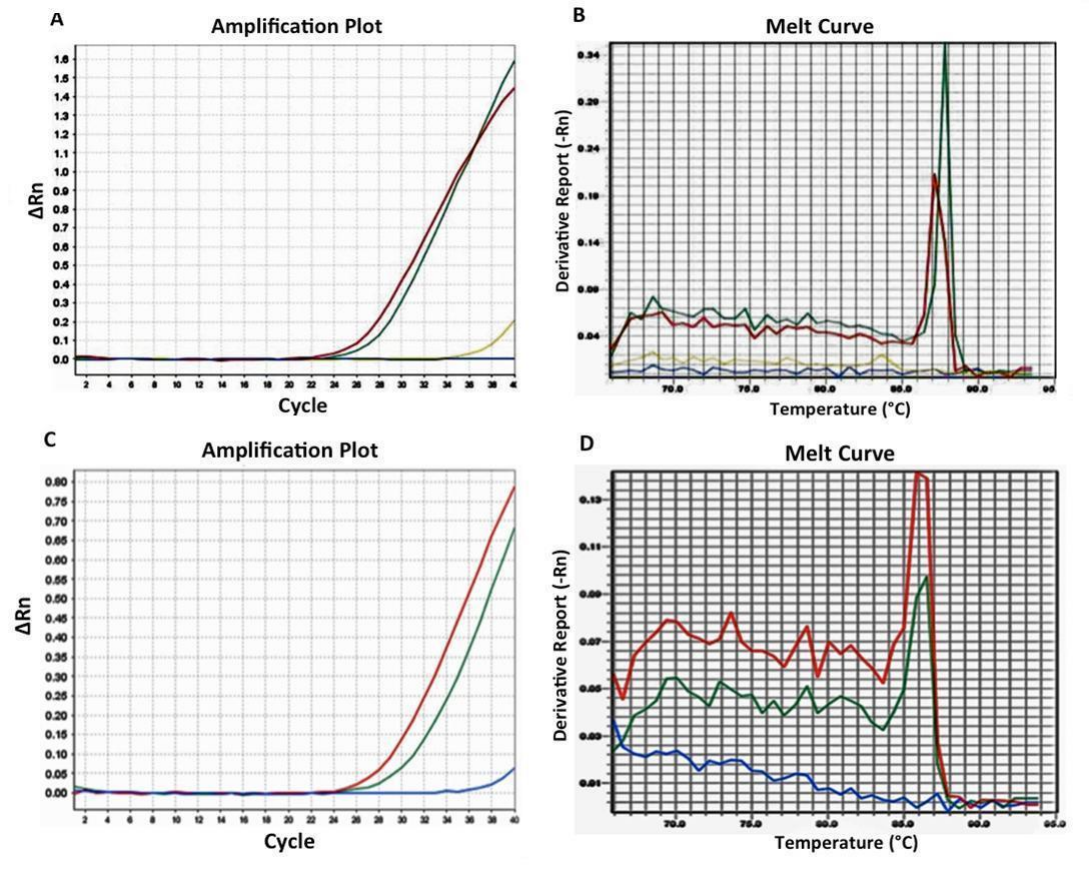
amplification plots (panels A and C) and the corresponding melting curve (panels B and D)

A total of 248 HIV-infected patients (66 women and 182 men) were enrolled and admitted to this prospective HLA-B*57.01 screening for the prevention of ABC-correlated HSR. 215 individuals were Italians from Southern Italy, 33 were non-EU. None of the patients had previously received a treatment with abacavir. HLA-B*57.01 frequency values were similar in both Italians (2.8%) and non-EU (3.0%). In Figure 1 amplification plots (panels A and C) and the corresponding melting curve (panels B and D) were showed.

## IV. DISCUSSION

Since 1998 abacavir has been used by almost 1 million HIV-infected patients [9]. This antiretroviral agent is generally well tolerated, but some patients develop an immunologic reaction, indicated as ABC-HSR, usually within the first 6 weeks of treatment. ABC-HSR is a relatively dose-independent adverse reaction generally characterized by fever, rash, gastrointestinal and/or respiratory symptoms [10]. It may be threatening and in some cases can cause death, especially in the case of re-challenge reaction due to ABC reintroduction. Moreover, the ABC-HSR diagnosis is complicated by the fact that there is a lack of specific signs and symptoms and, notably, the concomitant use of other antiretroviral drugs can be responsible for overlapping adverse events [11,12].

Because only a minority (2–9%) of patients develops this immunologic reaction, it was hypothesized that individual characteristics could also play an important role. In addition, some epidemiologic studies identified racial differences responsible for the different risk to develop ABC-HSR. From 2002, worldwide studies have reliably showed that HLAB*57.01 is important to predict the hypersensivity reactions to abacavir [4,5,7,13].

Interestingly, while a high frequency of HLAB*57.01 allele was found in white people, the same was not observed in black ones [14]. On the other hand, an almost 100% sensitivity of HLA-B*57.01 as a marker to predict the individual patient’s risk for ABC-HSR was confirmed in both US white and black people, suggesting the existence of a real clinical utility of this genetic screening in both races [15].

The prevalence of HLA-B*57.01 carriers greatly varies (from 2 to 9%) in different ethnic/geographic groups [16]. We found an HLA-B*57.01 frequency of 2.8% in patients coming from Southern Italy.

For what concerning HLA-B*57.01 carriers in the group of non-EU patients, we found an allelic frequency of ~3%, a value that is quite similar to that obtained for the Italian group. However, it is worth to note that this value is referred to a mixed group that includes individuals coming from several non-EU member countries.

It has been estimated that in the absence of a genetic prospective screening, ABC-HSR might affect ca. 6% of patients. As a consequence, the pharmacogenetic screening for HLA-B*57.01 is now recommended before starting ABC therapy in both adult and adolescent HIV-infected patients [17].

Regrettably, in spite of the fact that HLA-B*57.01 genotyping is a cost effective analysis that is useful in preventing ABC-HSR [18,19], it is still an underused clinical practice.

Since the HLA locus is extremely polymorphic, it is difficult to discriminate HLA-B*57.01 from related alleles. In recent years several molecular genetic methodologies, such as sequence specific amplification [20], allele-specific polymerase chain reaction (AS-PCR)/melting curve [21] and many others, have been proposed. In our laboratory we have successfully used, and continue to utilize, a Real-Time PCR technique with Sybr-Green for the identification of HLA-B*57.01 carriers.

The Real-Time PCR was recognized as a very fast (the reaction is complete in 2–3h) and cost-effective screening technique with an average reagent cost of about 16 Euros [22]. Our experience confirm that it is a quick and reliable technique to screen HLAB*57.01 carriers with high specificity, sensitivity and reproducibility.

## V. CONCLUSIONS

Abacavir is one of the brightest examples of “translation” of basic research studies into clinical practice.

HLA-B*57.01 screening before abacavir administration has been shown to be of great clinical utility, and it is now officially recommended in clinical guidelines and routinely performed in most Western countries.

Although genotyping should not substitute the clinical vigilance, it greatly reduces the incidence of ABC-HSR by identifying, before starting the therapy, patients at high risk of developing such adverse reaction. We hope that our laboratory experience, here reported, may positively contribute to the clinical use of this specific genotyping in the more general framework of pharmacogenetic mission to encourage personalized diagnosis and therapy.
